# Epidemiology of tuberculous lymphadenitis in Denmark: A nationwide register-based study

**DOI:** 10.1371/journal.pone.0221232

**Published:** 2019-08-15

**Authors:** Victor Dahl Mathiasen, Andreas Halgreen Eiset, Peter Henrik Andersen, Christian Wejse, Troels Lillebaek

**Affiliations:** 1 International Reference Laboratory of Mycobacteriology, Statens Serum Institut, Copenhagen, Denmark; 2 Department of Infectious Diseases, Aarhus University Hospital, Aarhus, Denmark; 3 Center for Global Health (GloHAU), Department of Public Health, Aarhus University, Aarhus, Denmark; 4 Department of Infectious Disease Epidemiology and Prevention, Statens Serum Institut, Copenhagen, Denmark; Agencia de Salut Publica de Barcelona, SPAIN

## Abstract

**Background:**

Tuberculous lymphadenitis (TBLA) is the most common extrapulmonary manifestation of tuberculosis (TB), often claimed to be reactivation. We aimed to describe the epidemiology of TBLA in Denmark, as it has not previously been investigated specifically although extrapulmonary TB has been associated with an increased long-term mortality and delays in the diagnosis.

**Methods:**

Register-based study of all patients notified with TBLA in Denmark from 2007 through 2016 utilizing six different nationwide registers. Patients were identified through the national TB surveillance register, and the diagnosis evaluated based on microbiology, pathology and/or clinical assessment.

**Results:**

In total, 13.5% (*n* = 489) of all TB patients in Denmark had TBLA with annual proportions from 9.4 to 15.7%. Most patients were immigrants between 25–44 years. Incidence rates ranged from as high as 1,014/100,000 for Nepalese citizens to as a low as 0.06/100,000 for Danes. Danes had a significant higher median age and significant more risk factors and comorbidities, as well as an increased overall mortality, compared with immigrants (*p*<0.05). A significant and much higher proportion of unique MIRU-VNTR genotypes were seen among TBLA patients compared to other TB manifestations.

**Conclusion:**

In Denmark, TBLA is a common manifestation of TB, especially in young immigrants from high-incidence countries. In Danes, it is a rare disease manifestation and associated with higher morbidity and mortality. To our knowledge, this is the first study suggesting that TBLA is predominantly associated with reactivation of latent TB infection based on genotyping although this remains to be clarified.

## Introduction

From 2002 through 2011, the proportion of extrapulmonary tuberculosis (EPTB) has increased in the European Union [1]. This proportional increase might continue with intensified targeting of pulmonary TB (PTB) and in the light of the recent European migrant crisis as well as increasing globalization.

The most common EPTB manifestation is tuberculous lymphadenitis (TBLA) which often affects the cervical lymph nodes [2]. Interestingly, TBLA is predominantly seen in females and in low-incidence countries, mainly among immigrants [3]. Typically, TBLA is attributed to late tuberculosis (TB) disease caused by reactivation of latent tuberculosis infection (LTBI), although this association has not been validated to our knowledge and the underlying mechanism remain unclear [4]. In TB low-incidence countries such as Denmark, a high index of suspicion is required to diagnose TBLA. Often, the clinicians lack experience and awareness of this disease manifestation as TBLA is rare. Symptoms are vague and the potential differential diagnoses many [3]. Consequently, this may result in delays in diagnosis which potentially increases the morbidity and mortality for the affected patients and constitutes sources of *Mycobacterium tuberculosis* (Mtb) transmission in undiagnosed patients with concurrent pulmonary infection [5,6]. In a recent systematic review, we demonstrated that TBLA patients have a mean health care delay of up to as many as 94 days in low-incidence, high-income countries [5]. Furthermore, EPTB has been associated with an increased long-term mortality [7,8].

In Denmark in 2017, 275 patients were notified with TB of which 22.2% had EPTB [9]. Approximately two-thirds of these had TBLA, corresponding to 14.5% of all notified TB patients in the country. Nevertheless, TBLA epidemiology in Denmark has never been investigated systematically.

We aimed to describe the epidemiological characteristics of TBLA patients in Denmark including demography, molecular epidemiology, treatment outcome, comorbidity, and mortality.

## Materials and methods

### Study design

We conducted a nationwide register-based study of all patients notified with TBLA in Denmark from 2007 through 2016. The study was performed and reported in accordance with the STROBE (Strengthening the Reporting of Observational Studies in Epidemiology) guidelines (S1 Table) [10].

### Study setting

Denmark is a high-income country with a low overall TB incidence (4.8/100,000 citizens in 2017) and a low human immunodeficiency virus (HIV) prevalence (70/100,000) [9,11]. Citizens have free access to health care and TB-medications, and Bacille Calmette Guerin vaccination was phased out of the childhood immunization programme during the early 1980’s.

All mycobacteria diagnostics in Denmark are centralized at the International Reference Laboratory of Mycobacteriology (IRLM) at Statens Serum Institut (SSI), Copenhagen, where clinical samples from all potential TB patients are examined with fluorescence microscopy, polymerase chain reaction (PCR), culture, and genotyping techniques. Since 2007, MIRU-VNTR genotyping has been performed on all Mtb culture positive cases [12]. In addition, all patients diagnosed with TB are notified to the Department of Infectious Disease Epidemiology and Prevention (DIDE), SSI. Notification of TB is mandatory for both the clinician initiating treatment and for the IRLM.

### Study material and population

**Fig 1:**
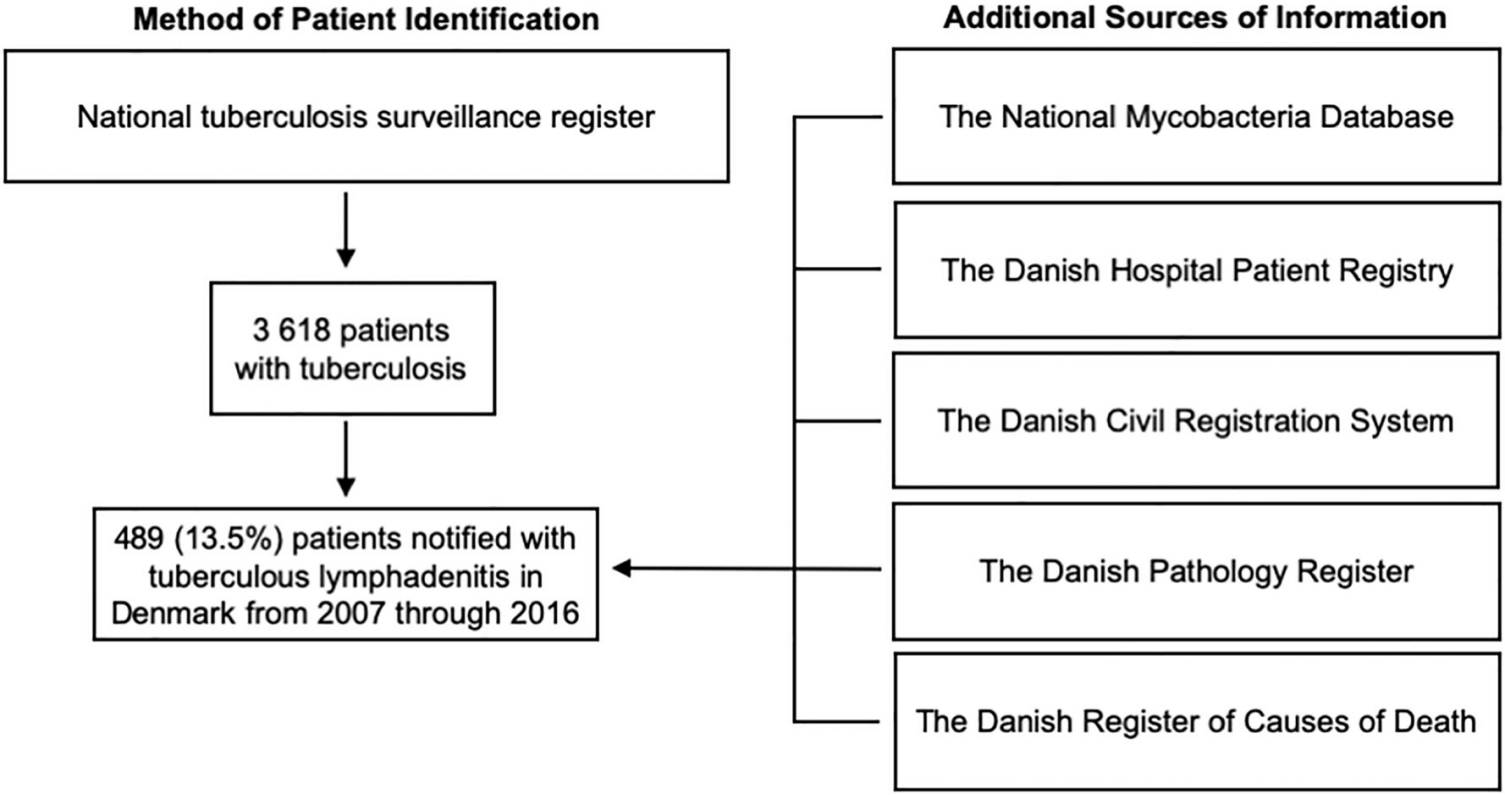
Danish registers used. Overview of the different nationwide registers used for patient identification and data extraction. All collected data relied on notification from the individual clinician(s) managing the patient.

Patients were identified through the national TB surveillance register and linked to other databases through the Civil Registration Number (CPR), a unique personal identifier given to all citizens in Denmark (Fig 1). These registers contains nationwide data on mycobacterial examination results, patient demographics and hospitalization information, comorbidities, pathological examinations, and cause(s) of death, among others [13–16].

We included patients of all ages notified with TBLA, including patients with concomitant TB in other organs and a previous history of TB. The diagnosis was based on either microbiology, pathology or clinical assessment. Infection with mycobacteria other than Mtb complex bacteria was an exclusion criterion. Case definition and treatment outcome were defined according to ECDC and WHO criteria [17,18]. Confirmed cases were either culture-positive or PCR and microscopy-positive. Probable cases were PCR or microscopy positive or had pathology suggestive of TB. Finally, possible cases were based on a clinical assessment. Countries were categorized by regions according to WHO definitions (Listed in S2 Table) [19].

### Statistical analysis

Contingency tables were constructed for patient characteristics and treatment outcome and survival by Danes, immigrants and overall. Immigrants included patients born outside Denmark and their children born in Denmark and Greenlanders living in Denmark. The sex distribution was assessed using a binomial test, while other variables were compared using Fisher’s exact test. We calculated a modified Charlson Comorbidity Index (CCI) score as a measure of comorbidity based on 19 conditions classified by *International Diseases Classification* (ICD-8/10) codes from 1977 until the date of disease notification [20]. Annual incidence rates were calculated using the number of TB patients in our data extraction as reported by the DIDE, SSI, and the Danish population and subpopulations (i.e. immigrants and Danish-born) reported by Statistics Denmark [21]. The date of first symptoms reported by the patient, if available, or the date of disease notification, was defined as the year of diagnosis. A non-parametric test (Spearman’s rank sum correlation test) was used to evaluate changes in the TBLA/all TB-ratio during the study period. All statistical tests were conducted using Stata/IC 15.1 (StataCorp, College Station, TX, USA), presented with two-sided 95% confidence interval and with a significance level of *p* <0.05, where appropriate.

### Ethics

The study was approved by the Danish Data Protection Agency (1-16-02-73-17) and Danish Patient Safety Authority (3-3013-2108/1). As the study was conducted with the use of register-based data and without any patient contact, or with the use of individual patient records, approval from The Central Denmark Region Committees on Health Research Ethics was waived after our inquiry (1-10-72-189-16). Patient information was pseudo-anonymized and de-identified prior to data analysis and stored under existing legislation. All data presented in public is anonymized, i.e. no single participant or groups hereof is recognizable.

## Results

### Patient characteristics

**Fig 2:**
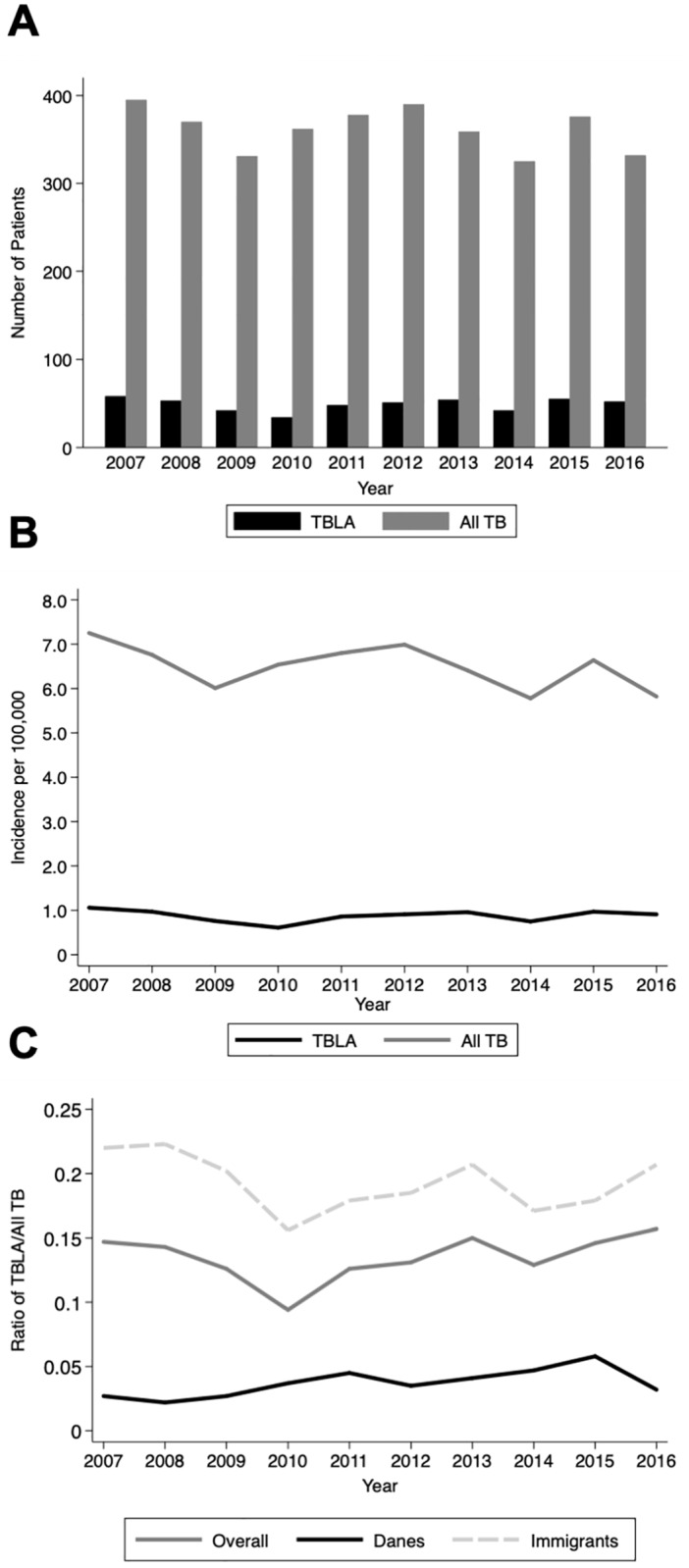
Incidence of tuberculous lymphadenitis. **A)** Number of patients notified with tuberculous lymphadenitis (TBLA) and all notified tuberculosis (TB) cases in the national TB register from 2007 through 2016, **B)** Corresponding incidence rates per 100,000 citizens and **C)** Overall ratio of TBLA/all TB and for immigrants and Danes respectively.

During the study period, 3,618 TB-patients were notified to DIDE of which 13.5% (*n* = 489) were TBLA patients (Fig 2). Annual proportions of TBLA ranged from 9.4–15.7%. Ninety-one percent of the TBLA patients were immigrants, 54.8% between the age of 25 and 44 years (Table 1). Fifty-three percent were females (*p* = 0.278). Almost nine out of ten patients had isolated TBLA, while the most frequent site of concurrent infection was pulmonary (10.8%). Danes were significantly older, had significant more risk factors and higher CCI scores compared with immigrants (*p*<0.05) (Table 1). Data for CCI score calculation was available for *n* = 458/484. The cases without comorbidity data were younger male immigrants with a temporary personal identifier only (asylum seekers, tourists etc.). In total, 24 patients had a temporary personal identifier only. Information about HIV status was available for only 8.2% of the patients.

**Table 1:** Patient demographics. Demographic characteristics and Charlson comorbidity index (CCI) scores for tuberculous lymphadenitis patients in Denmark from 2007 through 2016.

	Overall		Danes		Immigrants		
Patient characteristics	n/N	%	n/N	%	n/N	%	*p*-value^a^
Patients with TBLA	489	100.0	45	9.2	444	90.8	
Females	257/489	52.6	23/45	51.1	234/444	52.7	0.876
Age median, years (IQR)	32 (24–43)		47 (30–66)		32 (25–41)		
Age groups, years							0.000
< 15	27/489	5.5	5/45	11.1	22/444	5.0	
15–24	84/489	17.2	-	-	84/444	18.9	
25–44	268/489	54.8	15/45	33.3	253/444	57.0	
45–64	85/489	17.4	13/45	28.9	72/444	16.2	
>65	25/489	5.1	12/45	26.7	13/444	2.9	
Previous episode of TB	27/489	5.5	4/45	8.9	23/444	5.2	0.289
One or more concomitant disease site(s)^b^	67/489	13.7	5/45	11.1	62/444	14.0	0.820
One or more risk factor(s)^c^	19/489	3.9	7/45	15.6	12/444	2.7	0.001
Known HIV status	40/489	8.2	4/45	8.9	36/444	8.1	1.000
Infected	15/40	37.5	2/45	50.0	13/36	36.1	
Uninfected	25/40	62.5	2/45	50.0	23/36	63.9	
CCI score							0.000
0	345/458	75.3	16/45	36.4	329/414	78.3	
1–2	64/458	14.0	12/45	27.3	52/414	12.6	
≥3	49/458	10.7	16/45	36.4	33/414	8.0	

Abbreviations: TBLA, tuberculous lymphadenitis. IQR, interquartile range. TB, tuberculosis. HIV, human immunodeficiency virus. CCI, charlson comorbidity index.

^a^*p*-values for comparison of Danes and immigrants using Fisher’s exact test.

^b^Pulmonary, bone-joint, pleural, abdominal, skin, urogenital, or other.

^c^Risk factors notified: Hepatitis C infection, alcohol abuse, homelessness, diabetes mellitus, renal insufficiency, TNF-alpha-inhibitor treatment, chronic lymphocytic leukaemia.

### Incidence of TBLA

**Fig 3:**
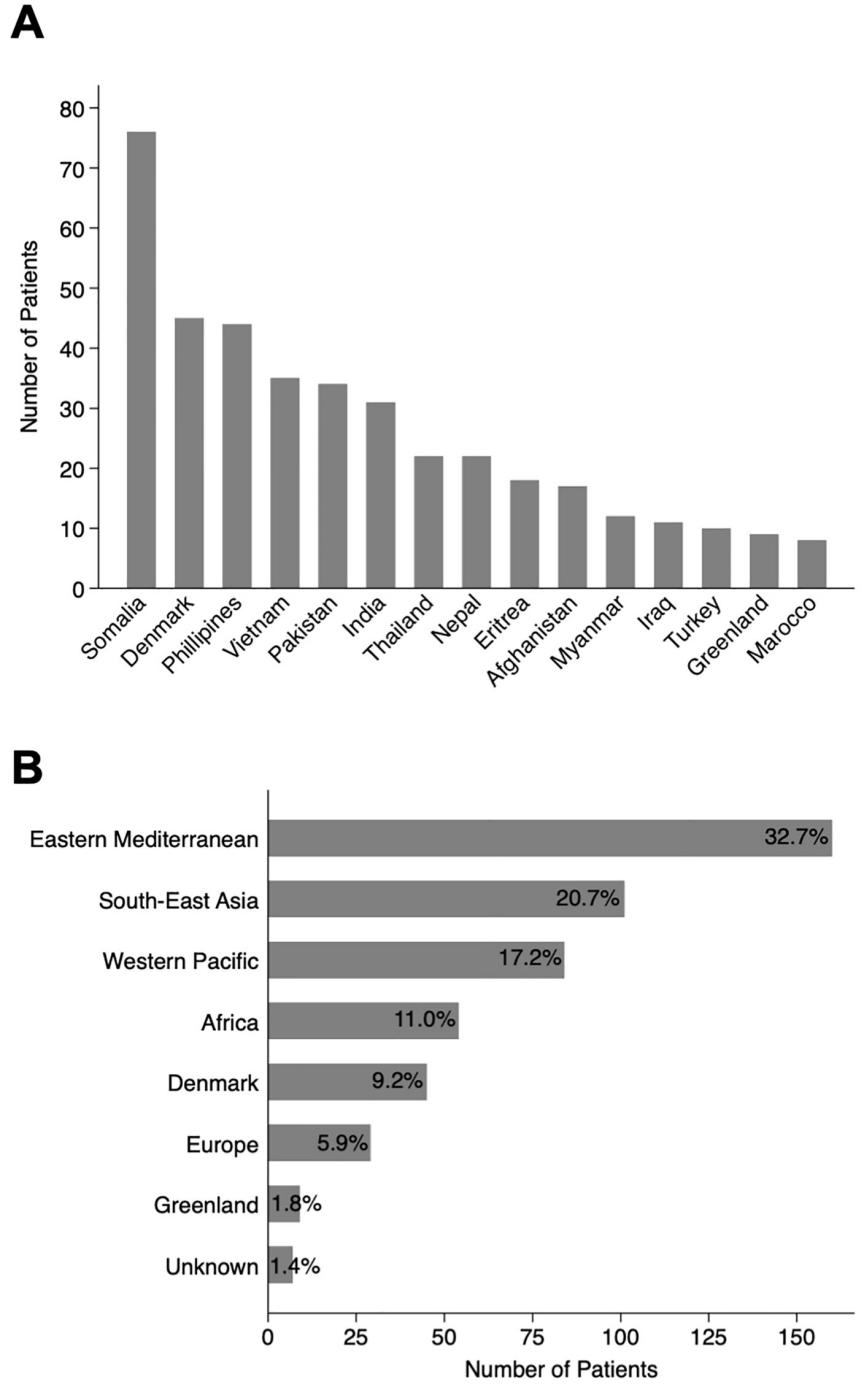
Country and regions of origin. **A)** The number of tuberculous lymphadenitis patients from the 15 most frequent countries of origin, **B)** Patient origin by WHO regions in percentages. Of notice, Somalia and the northern parts of Africa is by WHO included in the Eastern Mediterranean region [19]. Classification of countries by WHO regions is available in the S2 Table.

Fifty-three nationalities were represented among the 489 patients with most originating from the Eastern Mediterranean and South-East Asia regions (Fig 3). For immigrants, the five most frequent countries of origin were Somalia (*n* = 76), Philippines (*n* = 44), Vietnam (*n* = 35), Pakistan (*n* = 34), and India (*n* = 31).

Nine Greenlanders represented 1.8% of TBLA patients, while accounting for 15.2% (*n* = 551) of all TB patients in Denmark during the same period. The highest overall incidence rate (IR) of TBLA was in 2007 with 1.06/100,000 citizen, while the lowest was in 2010 with 0.61/100,000 (Fig 2). Danes had IRs ranging from 0.06–0.12/100,000. For countries where ten or more patients originated, the five highest IRs were Nepal 1014, Eritrea 722, Myanmar 142, India 108, and Philippines 70/100,000, respectively.

There was no significant increasing or decreasing trend in the proportion of TBLA over the last decade (Spearman’s rho = 0.370, *p* = 0.293).

### Diagnostic criteria and molecular epidemiology

According to ECDC case definitions [17], 67.3% of all cases were confirmed and 18.8% probable based on microbiological and pathological examination of lymph nodes, while 13.9% were possible based on clinical assessment. Almost all culture-confirmed cases (*n* = 324) were infected with Mtb. In total, 7.4% were drug resistant, only 0.6% multi-drug resistant. For 320 patients, at least one Mtb isolate was MIRU-VNTR genotyped distributed on 279 different genotypes represented, of which 74.9% (*n* = 209) were unique and not seen in other patients in Denmark during the study period. The most frequent genotypes were 1112–15 (*n* = 11) followed by 1557–32 (*n* = 7) while 18 genotypes were represented 2–4 times. A much higher proportion of unique MIRU-VNTR genotypes were seen among patients with TBLA compared with other manifestations of TB (i.e. compared to all other TB cases during the study period) (65.3% vs. 27.4%, *p*<0.05).

### Treatment outcome and mortality

Almost one-fourth of the notified patients had an unknown treatment outcome (Table 2). Among those with a reported outcome, most patients (64.6%) were notified as having completed treatment, but a significant higher proportion of immigrants had an unknown treatment outcome compared with Danes, and conversely significantly more Danes were notified with completed treatment (*p*<0.05). Survival data from The Danish Civil Registration System was available for *n* = 460/484 of the patients and significant more Danes (*n* = 7/44, 15.9%) died within five years of treatment initiation compared with immigrants (*n* = 8/416, 1.9%) (*p*<0.05). TB was the cause of death in two of these cases. The 24 cases without survival data were younger male immigrants with a temporary personal identifier only.

**Table 2:** Treatment outcome. Treatment outcome and all-cause mortality data for patients with tuberculous lymphadenitis in Denmark from 2007 through 2016.

	Overall		Danes		Immigrants		
Treatment outcome^a^	n/N	%	n/N	%	n/N	%	*p*-value^b^
Cured	17/489	3.5	1/45	2.2	16/444	3.6	1.000
Completed	316/489	64.6	39/45	86.7	277/444	62.4	0.001
Dead	5/489	1.0	2/45	4.4	3/444	0.7	0.069
Failure	10/489	2.0	1/45	2.2	9/444	2.0	1.000
Transfer	24/489	4.9	-	-	24/444	5.4	0.152
Unknown	117/489	23.9	2/45	4.4	115/444	25.9	0.001
**All-cause mortality**							
Dead within 5 years of treatment initation	15/460	3.3	7/44	15.9	8/416	1.9	0.000

^a^Based on treatment outcome definitions by WHO [18].

^b^*p*-values for comparison of Danes and immigrants using Fisher’s exact test.

## Discussion

In this nationwide register-based study covering 10 years, TBLA was the most common EPTB manifestation as also described previously [22]. The vast majority of TBLA patients were young immigrants with no other concurrent TB manifestations. One out of ten TB patients during this period had TBLA. We did not observe an increasing incidence from 2007 through 2016. TBLA was a rare disease manifestation for Danish-born TB patients, who were significantly older and had significantly more risk factors and comorbidities, and an increased mortality within five years of treatment initiation, compared with immigrants. Finally, we observed a high percentage of unique MIRU-VNTR genotypes among TBLA patients (75%).

The predominance of TBLA observed in immigrants, as previously described for paediatric TB patients [23], cannot solely be explained by health inequities, as health care is free in Denmark. However, the higher prevalence of TBLA among immigrants could reflect “late TB” following reactivation of LTBI acquired prior to migration to Denmark [24]. TBLA has often been associated with Mtb reactivation rather than primary TB [4], but to our knowledge, this association has not been documented. Using MIRU-VNTR, our study supports this theory, or at least renew speculations about this association, as suggested by the high proportion of unique genotypes seen among TBLA patients compared with other manifestations of TB (65.3% vs. 27.4%, *p*<0.05). It is also supported by the fact that a history of TB was recorded for 5.5% of the TBLA patients compared with 1.8% of all TB-patients in Denmark in another study [25]. Unfortunately, we were not able to conclude this association between TBLA and reactivation as dates of arrival in Denmark for immigrants and information about visits to high-incidence settings were not available. Yet, we find this the most plausible explanation of the high number of unique genotypes seen in TBLA patients, and regardless, an indicator of imported TB. Previous studies from Denmark have estimated that up to three-quarters of immigrant TB may be caused by reactivation of imported disease [24]. So regardless, increased targeting of LTBI, such as screening of immigrants from TB endemic regions and following treatment in infected, might potentially prevent the majority of TBLA cases [26]. Future molecular epidemiology studies are warranted and could improve our understanding of Mtb transmission and patterns of reactivation in TB and TBLA but is limited by the lack of molecular data from countries of origin.

Among Danish-born TB patients in general, TB has often been associated with specific risk factors, e.g. social marginalization or reactivation of LTBI due to increasing age [27,28]. The higher five-year mortality seen among Danes in our study could partly be explained by a higher median age, but probably also reflects that TB in Danes more often is seen in vulnerable and hard-to-reach groups such as homeless or drug abusers [27]. For the immigrant TBLA patients, a more recent and intensive exposure in the their country of origin most likely explains their younger age [28] but also reflects that individuals migrating are often young or middle-aged [29], as migration may be physically demanding.

As many as 53 different nationalities were represented in the cohort underlining the complexity of the TB epidemiology in Denmark. Most patients originated from the Eastern Mediterranean and South-East Asia WHO regions [19] with a majority from Somalia. Very few patients (1.8%) originated from Greenland, a population group associated with a high burden of TB [30]. However, EPTB has previously been described to be less common in Greenlanders [31]. Similarly, TBLA only corresponded to 3.7% of all TB among Danish-born during the study period while accounting for 19.4% of all TB among immigrants. In contrary to Greenlanders, EPTB has also previously been shown to be more much common among foreign-born TB patients in Denmark [31].

As opposed to previous reports [1,3], we did not find an increasing incidence in TBLA during the study period, nor did we find a significant difference in the prevalence of TBLA among females and males. Our study was very heterogeneous in terms of ethnic distribution and this, together with differences in biological, social, environmental, and/or cultural compositions, may explain varying findings in studies on TBLA [32,33]. Differences between TBLA and PTB patients, e.g. the overweight of females in EPTB, and other variations in the clinical presentation of TB, is in all probability influenced by a complex interaction between both host and bacterial factors. For instance, specific lineages of Mtb have been associated with TBLA [34]. Nonetheless, our assumption is, despite the lack of information on timing of disease, that TBLA is associated with reactivation of LTBI rather than ongoing transmission of a specific circulating TB genotype as a substantial amount of the genotyped Mtb isolates were unique based on MIRU-VNTR.

Monitoring of treatment outcome in TB control programmes is essential [35], and our study shows that monitoring and notification of treatment outcome could be improved for TBLA patients in Denmark as almost one-fourth was notified with an unknown treatment outcome. Most patients were registered as “treatment completed” or “unknown”, but we find it unlikely that 17 patients met the WHO treatment outcome criteria for cured, as four of these had isolated TBLA, which would require at least two negative biopsies during follow-up, one of these in the last month of treatment (i.e. these may be misclassified). Treatment outcome reporting is unfortunately not mandatory in Denmark and the quality of reports not evaluated. However, some internal validation is performed to minimize misclassification.

An improved focus on notification of treatment outcome is warranted to optimize TB disease surveillance in Denmark. Furthermore, a greater awareness as well as further studies of the diagnostic and clinical features of TBLA are needed to improve the clinical management and our current understanding. In most low-incidence countries, TBLA is rare and the clinician not used to diagnose this disease manifestation. Our recent review suggests that TBLA patients experience delays in both presentation, diagnosis and treatment [5]. The diagnosis may be complicated due to difficulties in obtaining good samples for microbiological examination [36], e.g. only 66.3% of all cases were culture-confirmed in our study. These observations are possibly attributable to most manifestations of EPTB.

Our study has some limitations. The largest limitation is the lack of time-interval from arrival of immigrants in Denmark to the diagnosis of TB as this limits the analysis of the association between TBLA and reactivation. Further, the study design limited systematic data collection, e.g. data for CCI score and mortality was not available for 26 and 24 patients, respectively. These were all immigrants, nine out of ten with a temporary personal identifier only, approx. 80% were males and they were on average six years younger compared with the rest of the cohort. The lack of clinical data and/or a control group, e.g. PTB patients, is another limitation. Further, another limitation is that the TB surveillance register does not have complete registration of all clinical data such as symptoms, chest radiograph, risk factors (e.g. HIV status), source(s) of infection, previous episodes of TB, treatment outcome, and accurate dates for all patients, as these information relies on notification from the individual clinicians. Information on HIV status was very limited and was only available for 8.2% although increasing over the last study years. Despite HIV-TB is rare in Denmark [37], we must emphasize that HIV status should be determined in all patients.

Despite limitations, the large sample size and nationwide inclusion of patients with TBLA are strengths underlined by the limited sample sizes seen in most other studies from low-incidence settings [3].

In summary, TBLA is the most frequent EPTB manifestation in Denmark predominantly seen in young immigrants and more rarely in vulnerable Danish-born patients. We found no trend in the proportion of TBLA during the last decade. Most Mtb isolates harboured unique Mtb genotypes suggesting that TBLA could be associated with reactivation of LTBI. Consequently, if this is truly an association, future targeting of LTBI in risk groups could potentially reduce the number of TBLA cases in Denmark, if we want to eliminate TB prospectively.

## Supporting information

S1 Table STROBE statement—Checklist of items that should be included in reports of observational studies. (PDF)

S2 Table WHO regions. Countries categorized by regions according to WHO definitions: https://www.who.int/about/who-we-are/regional-offices.
(PDF)
