# Supplementary material for: Epidemiology of tuberculous lymphadenitis in Denmark: A nationwide register-based study
Source: PLoS One. 2019 Aug 15;14(8):e0221232. doi: 10.1371/journal.pone.0221232 (PMC6695100; doi:10.1371/journal.pone.0221232)
Supplement: S2 Table — Countries categorized by regions according to WHO definitions: https://www.who.int/about/who-we-are/regional-offices. (PDF) [file pone.0221232.s002.pdf]

S2 Table. WHO regions. Countries categorized by regions according to WHO definitions: https://www.who.int/about/who-we-are/regional-offices

African Region	Americas	South-East Asia	Europe	Eastern Mediterranean	Western Pacific
Algeria	Anguilla	Bangladesh	Albania	Afghanistan	American Samoa
Angola	Antigua and Barbuda	Bhutan	Andorra	Bahrain	Australia
Benin	Argentina	Democratic People's Republic of Korea	Armenia	Djibouti	Brunei Darussalam
Botswana	Aruba	India	Austria	Egypt	Cambodia
Burkina Faso	Bahamas	Indonesia	Azerbaijan	Iran (Islamic Republic of)	China
Burundi	Barbados	Maldives	Belarus	Iraq	China, Hong Kong SAR
Cabo Verde	Belize	Myanmar	Belgium	Jordan	China, Macao SAR
Cameroon	Bermuda	Nepal	Bosnia and Herzegovina	Kuwait	Cook Islands
Central African Republic	Bolivia (Plurinational State of)	Sri Lanka	Bulgaria	Lebanon	Fiji
Chad	Brazil	Thailand	Croatia	Libya	French Polynesia
Comoros	British Virgin Islands	Timor-Leste	Cyprus	Morocco	Guam
Congo	Canada		Czechia	Oman	Japan
Cote d'Ivoire	Cayman Islands		Denmark	Pakistan	Kiribati
Democratic Republic of the Congo	Chile		Estonia	Qatar	Lao People's Democratic Republic
Equatorial Guinea	Colombia		Finland	Saudi Arabia	Malaysia
Eritrea	Costa Rica		France	Somalia	Marshall Islands
Eswatini	Cuba		Georgia	Sudan	Micronesia (Federated States of)
Ethiopia	Dominica		Germany	Syrian Arab Republic	Mongolia
Gabon	Dominican Republic		Greece	Tunisia	Nauru
Gambia	Ecuador		Greenland	United Arab Emirates	New Caledonia
Ghana	El Salvador		Hungary	West Bank and Gaza Strip	New Zealand
Guinea	Grenada		Iceland	Yemen	Niue
Guinea-Bissau	Guatemala		Ireland		Northern Mariana Islands
Kenya	Guyana		Israel		Palau
Lesotho	Haiti		Italy		Papua New Guinea
Liberia	Honduras		Kazakhstan		
			Kyrgyzstan		

Madagascar<br>Malawi<br>Mali<br>Mauritania<br>Mauritius<br>Mozambique<br>Namibia<br>Niger<br>Nigeria<br>Rwanda<br>Sao Tome and Principe<br>Senegal<br>Seychelles<br>Sierra Leone<br>South Africa<br>Togo<br>Uganda<br>United Republic of Tanzania<br>Zambia<br>Zimbabwe	Jamaica<br>Mexico<br>Montserrat<br>Netherlands Antilles<br>Nicaragua<br>Panama<br>Paraguay<br>Peru<br>Puerto Rico<br>Saint Kitts and Nevis<br>Saint Lucia<br>Saint Vincent and the Grenadines<br>Suriname<br>Trinidad and Tobago<br>Turks and Caicos Islands<br>United States of America<br>Uruguay<br>Venezuela (Bolivarian Republic of)		Latvia<br>Lithuania<br>Luxembourg<br>Malta<br>Monaco<br>Netherlands<br>North Macedonia<br>Norway<br>Poland<br>Portugal<br>Republic of Moldova<br>Romania<br>Russian Federation<br>San Marino<br>Serbia & Montenegro<br>Slovakia<br>Slovenia<br>Spain<br>Sweden<br>Switzerland<br>Tajikistan<br>Turkey<br>Turkmenistan<br>Ukraine<br>United Kingdom of Great Britain and Northern Ireland<br>Uzbekistan		Philippines<br>Republic of Korea<br>Samoa<br>Singapore<br>Solomon Islands<br>Tokelau<br>Tonga<br>Tuvalu<br>Vanuatu<br>Viet Nam<br>Wallis and Futuna Islands
